# A revised instrument for the assessment of empathy and Theory of Mind in adolescents: Introducing the EmpaToM-Y

**DOI:** 10.3758/s13428-021-01589-3

**Published:** 2021-05-04

**Authors:** Christina Breil, Philipp Kanske, Roxana Pittig, Anne Böckler

**Affiliations:** 1 Leibniz-University Hanover, Schloßwender Straße 1, D-30159 Hanover, Germany; 2 Max Planck Institute for Human Cognitive and Brain Sciences, Leipzig, Germany; 3 Technische Universität Dresden, Dresden, Germany; 4 Julius-Maximilians-University of Wuerzburg, Wuerzburg, Germany

**Keywords:** Social cognition, Social understanding, Theory of mind, Mentalizing, Empathy, Adolescence, Development

## Abstract

Empathy and Theory of Mind (ToM) are two core components of social understanding. The EmpaToM is a validated social video task that allows for independent manipulation and assessment of the two capacities. First applications revealed that empathy and ToM are dissociable constructs on a neuronal as well as on a behavioral level. As the EmpaToM has been designed for the assessment of social understanding in adults, it has a high degree of complexity and comprises topics that are inadequate for minors. For this reason, we designed a new version of the EmpaToM that is especially suited to measure empathy and ToM in youths. In experiment 1, we successfully validated the EmpaToM-Y on the original EmpaToM in an adult sample (*N* = 61), revealing a similar pattern of results across tasks and strong correlations of all constructs. As intended, the performance measure for ToM and the control condition of the EmpaToM-Y showed reduced difficulty. In experiment 2, we tested the feasibility of the EmpaToM-Y in a group of teenagers (*N* = 36). Results indicate a reliable empathy induction and higher demands of ToM questions for adolescents. We provide a promising task for future research targeting inter-individual variability of socio-cognitive and socio-affective capacities as well as their precursors and outcomes in healthy minors and clinical populations.

**Supplementary Information:**

The online version contains supplementary material available at 10.3758/s13428-021-01589-3.

## Introduction

Today’s youth is more closely connected, better educated and more diverse than any generation before. These chances bring about novel challenges. In a globalized world, problems arise at a bigger scale and constructive cooperation is more important than ever. Two key social capacities are necessary for this endeavor. First, feeling for somebody or sharing someone’s affect, which is commonly referred to as “empathy” (de Vignemont & Singer, 2006; Oliver et al., 2018; Singer & Lamm, 2009), and second, the capability to represent another’s intentions and beliefs, commonly referred to as “theory of mind” (ToM) or “mentalizing” (Chris D. Frith & Frith, 2006). Even though these two concepts have many features in common, they can be clearly dissociated on a behavioral and on a neuronal level (Kanske et al., 2015; Schurz et al., 2020).

As early as on the first day of their lives, human infants spontaneously respond to hearing other infants’ cries (Martin & Clark, 1982). In parallel, the neural networks associated with empathy are subject to profound maturation processes until adulthood (Decety & Michalska, 2010). On a behavioral level, findings regarding age trends in empathy-related responding during adolescence are inconsistent: While Decety and Michalska (2010) found reduced intensity of pain perceptions in others with increasing age, other studies report an age-related increase in empathic responding, and some did not find any differences (Eisenberg et al., 2009).

There are profound inter-individual differences in adolescent empathy that remain stable across several decades (Allemand et al., 2015). These differences in empathy reflect on various other life domains: Impairments in empathic responding have been associated with aggression and criminal behavior across all age groups (Blair, 2018; van Hazebroek et al., 2017; van Zonneveld et al., 2017; Winter et al., 2017). In adolescents, empathy is negatively related to delinquency, bullying and externalizing problems—but positively related to numerous socially desirable characteristics, such as pro-social goals, social competence and supportive relationships (Eisenberg et al., 2009). Critically, the level of initial empathy as well as the degree and direction of development during adolescence predict inter-individual differences in social competence two decades later (Allemand et al., 2015) and an accumulation of adverse relationships in youths is considered an unspecific risk factor for psychopathologic development from adolescence to early adulthood (Adam et al., 2011). As such, adolescent empathy is not only a protective factor at the time being, but also an important resource for social functioning and mental health as an adult. Yet the literature on empathy development from ages 12–18 is limited and findings have been inconsistent (Eisenberg et al., 2009), indicating that further research in this area is urgently needed.

In a similar vein, research in children suggests a reliable development of ToM capacities during the first years of life (Hughes & Leekam, 2004; Wellman et al., 2001) with first attempts of spontaneous perspective-taking at the age of 7–15 months (Baillargeon et al., 2010; Kovacs et al., 2010) and a progressive understanding of more complex forms of mentalizing throughout adolescence (Devine & Hughes, 2013) and adulthood (Dumontheil et al., 2010). Difficulties in ToM performance have been linked to a variety of psychological disorders, such as depression, social anxiety disorder, autism spectrum disorder (ASD) and schizophrenia (Berecz et al., 2016; Bora et al., 2009; Leppanen et al., 2018; Washburn et al., 2016).

So far, most research has focused on the early childhood and preschool years (Baillargeon et al., 2010; Cadinu & Kiesner, 2000; Wellman et al., 2001), and on clinical populations with social deficits, for instance individuals with ASD (Altschuler et al., 2018; Baron-Cohen et al., 1985; Deschrijver et al., 2016) or schizophrenia (Bora et al., 2009; Frith & Corcoran, 1996). More recently, the neural underpinnings of mentalizing have received considerable attention, and new paradigms have been developed to investigate inter-individual variability in healthy adults (Baksh et al., 2018; Murray et al., 2017; Schurz et al., 2014, 2020). In striking contrast, very little attention has been devoted to ToM development, its precursors and its outcomes in healthy teenagers. Pioneer fMRI studies show that activity during mentalizing processes in frontal regions decreases from adolescence to adulthood (Blakemore et al., 2007; Sebastian et al., 2012; Wang et al., 2006), which could be indicative of synaptic reorganization processes in the prefrontal cortex (Blakemore, 2008). One recent study demonstrated that only from the age of 10–12 years onwards do children begin to understand that two people can represent the exact same information differently. This type of reasoning has been shown to be protective of serious behavior problems and social conflict in high school (Weimer et al., 2017). Strong interactions between peer acceptance and social understanding have been demonstrated in children (Banerjee et al., 2011; Hughes & Leekam, 2004) and pre-adolescents (Bosacki & Wilde Astington, 2001). A thorough investigation of social understanding in teenagers is therefore highly necessary.

Critically, the endeavor of assessing the development of empathy and ToM in youths demands measures that allow for an assessment of the full range of skills that are required to prosper in the adolescent social system. The false belief task (Wimmer & Perner, 1983) is widely regarded as the litmus test for ToM, but is already mastered by normally developing children from the age of four years on (Wellman et al., 2001), and even the more complex variations of this or related paradigms are usually at ceiling in healthy adults (but see Keysar et al. (2003)). These issues lower the chances of capturing variance and improvement in mental state representation in healthy adult and adolescent samples. In this light, the development of complex ToM measures, such as the Edinburgh Social Cognition test (Baksh et al., 2018) and the EmpaToM (Kanske et al., 2015), has been an important recent trend.

The EmpaToM is a promising tool, as it allows for simultaneous manipulation and assessment of empathy and ToM. By using naturalistic dynamic stimuli, the EmpaToM is akin to real-life situations and interactions, and its compatibility with physiological measures and imaging techniques allows for a full-range investigation of social cognition in healthy samples and clinical populations (Preckel et al., 2016). The task consists of short video sequences that depict an unknown person narrating an autobiographical episode. The episode is either of negative emotional valence, thereby eliciting an empathic response, or neutral as a control condition. Participant empathic tendency is derived from affect ratings after each video. ToM performance is measured by means of content-related questions on the previously seen video that either require mental perspective taking of the narrator (ToM), or not (nonToM). Hence, affect sharing and ToM are orthogonally manipulated in this task, comprising (i) negative and neutral videos for an assessment of subjective affect sharing of the participants and (ii) videos with or without a mentalizing component allowing for subsequent ToM questions and control questions on each story. The EmpaToM has been thoroughly validated, revealing specific brain-behavior relations for both capacities. Importantly, the task is sensitive for changes in social cognition across the adult lifespan (Reiter et al., 2017) and for plasticity induced by mental trainings (Trautwein et al., 2020). However, the EmpaToM in its present form is inappropriate for an assessment of empathy and ToM in adolescents for three reasons in particular. For one, this task encompasses several episodes that are inadequate for minors on an affective level. These episodes include war experiences, sexual and physical abuse, family tragedies and deadly accidents, and could lead to intolerable emotional distress in teenagers. Second, the EmpaToM has a high level of difficulty resulting from complex issues that are alluded to but not explicitly named in the videos, and from questions that require common knowledge that may only be acquired with age. Finally, the EmpaToM mainly entails “adult topics” that could be difficult to imagine for teenagers. While empathizing with other persons even when they are in situations one cannot easily relate to is a core competence of social understanding and should hence not lower ecological validity, the exclusive implementation of such unfamiliar topics could lead to low motivation or even negligence at task execution.

In summary, even though social cognition likely continues to develop beyond the well-studied hallmarks during childhood, a thorough understanding of the representation of other people’s minds in adolescents is still lacking. This gap is especially problematic because social competence is vital for a healthy and adaptive coming of age with intact peer relationships. Critically, the neglect of adolescent social cognition in research goes hand in hand with a shortage of appropriate tools for a comprehensive assessment of social understanding in healthy individuals of this age group.

We aimed to fill this gap by providing a new instrument for the assessment of empathic affect sharing and ToM in teenage samples. Our goal was to design a measure that allows for a full-range investigation of adolescent social understanding with inter-individual variability in a naturalistic setting. To this end, we created a version of the EmpaToM that is especially tailored to the abilities and needs of a younger age group, namely (i) eliciting sufficient inter-individual variance while being generally solvable by teens and (ii) age-appropriate content of the stories with (iii) younger narrators talking about issues that teenagers can more easily relate to. For our new instrument, the EmpaToM-Y, we kept the general design of the EmpaToM and developed new videos and questions that are less complex and more appropriate for adolescents.

Because the original EmpaToM has been extensively validated, we first behaviorally tested the EmpaToM-Y on the existing measure in an adult sample group (*N* = 61, experiment 1). We decided on this age group because the original EmpaToM is inappropriate for adolescents. We therefore conducted a second experiment (*N* = 36, experiment 2) in which we assessed the feasibility of our new instrument in a sample of adolescents. For further external validation in experiment 2, we added a standardized measure of self-reported empathy and ToM.

## Experiment 1

### Method

We report how the sample size was determined, all manipulations and measures that are collected and all data exclusions (Simmons et al., 2012). In experiment 1, we apply the EmpaToM-Y together with the existing EmpaToM in an adult sample to behaviorally validate the new measures.

#### Participants

Ninety-nine participants took part in experiment 1 in return for course credit or 10€ and completed an informed consent form. All participants were recruited via the participant database of the University of Wuerzburg, were fluent in German and reported normal or corrected to normal vision. We had to exclude the data of 18 participants because they reported being acquainted with one of the persons that was displayed in the videos, or because of language barriers. Due to technical difficulties with one of the testing computers, the data of 17 further participants was corrupt and could not be entered into the analysis. Of the remaining 64 participants, three data sets were removed due to implausibly high error rates in the ToM and nonToM questions (above 33%), leaving 61 participants (mean age = 28.7, SD = 8.88, range: 20–56; 47 females; 57 right-handed) for the final analysis. The present study is compliant with the ethical standards of the 1964 Declaration of Helsinki regarding the treatment of human participants in research and was approved by the local ethics committee.

#### Task

**Fig. 1:**
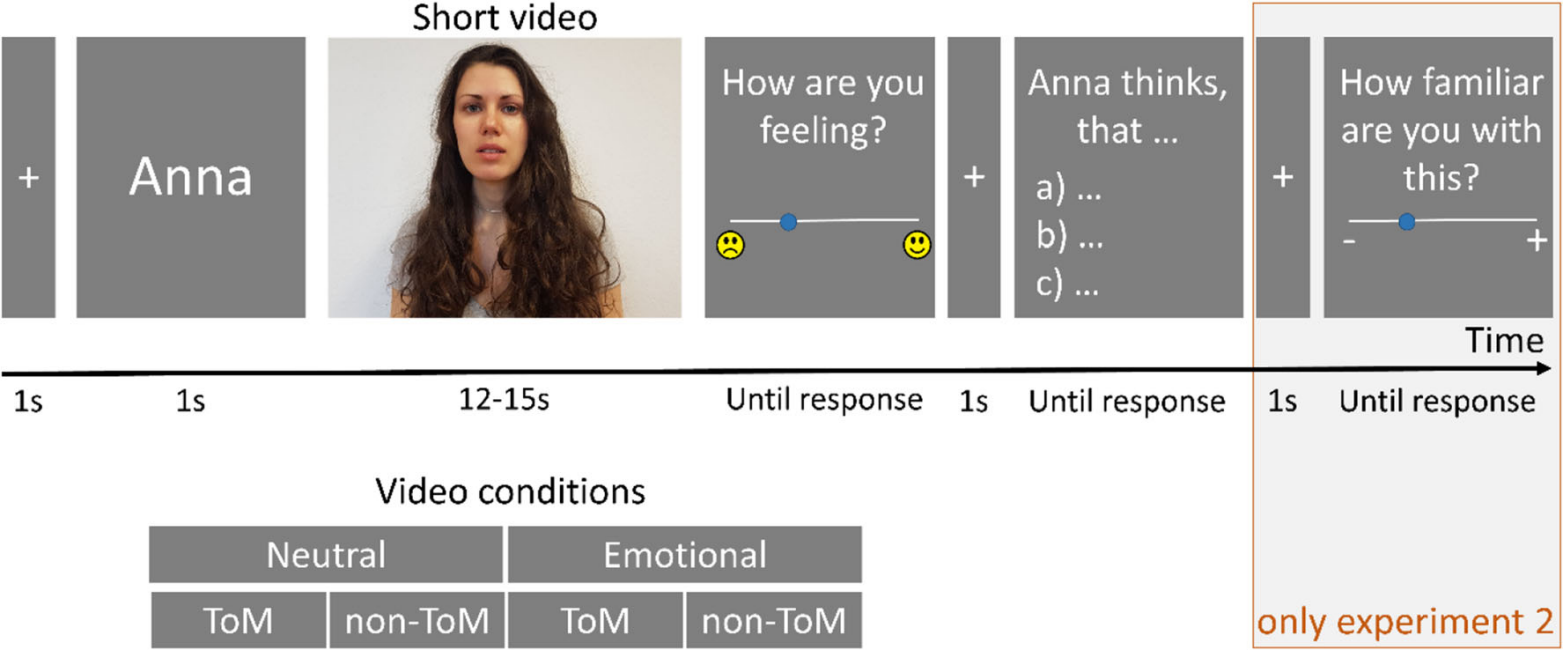
Trial sequence of experiment 1. Note. After a fixation cross and the name of the person in the video are displayed for 1 s each, a short video (12–15 s) is played. The video is followed by a rating scale measuring empathic affect and a multiple-choice question for ToM assessment or factual reasoning, both displayed until a response is made. In experiment 2, this was followed by a second rating question to assess familiarity with the situation in the video

The EmpaToM-Y is a German video-based task that simultaneously manipulates empathic affect sharing and ToM. Each trial started with a fixation cross (1 s) after which the name of the person who is speaking in the following video was displayed (1 s; Fig. 1). Each video lasted about 15 s and presented an unknown character allegedly recounting an autobiographical episode. The videos differed in terms of valence (neutral or negative) and ToM-affordance (ToM or nonToM). After each video, participants were required to rate their own emotional state on a rating scale ranging from negative to positive (affect rating). We derived a measure for the tendency to share others‘ affect (affect sharing tendency) by comparing the participants’ rating after negative versus neutral videos. The affect rating was followed by a multiple-choice question regarding the video content. Each question had three response options (one correct answer) that appeared in randomized order. The questions either entailed mental perspective-taking (ToM) or factual reasoning (nonToM). The EmpaToM-Y consisted of 40 trials (ten for each combination video valence and ToM requirement), with four videos per narrator (one per condition). Forty trials of the original EmpaToM task were presented intermixed with the 40 trials of the EmpaToM-Y in randomized order with a short break every 20 trials.

#### Stimuli

Twenty-four novel videos, six for each condition, were created specifically for the EmpaToM-Y. Each episode was designed to resemble an extract of a presumably longer dyadic conversation and was either neutral or emotionally negative, thus entailing experiences of disappointment, loss or regret. We took special care to avoid any age-inappropriate content, such as war experience, heavy violence or family drama, and to include more age-related topics like school life or peer group experiences. An example story and the corresponding question for each condition can be found in Appendix A. Six young amateur actors, three females and three males, were recruited for the shooting of the videos and were compensated with payment (10€/hour). One actress was Afro-German, the other five were Caucasian. The camera, light and audio settings were held constant throughout all of the videos. The film footage was cut to a length of 12–15 s per video and converted to MP4 using Windows Movie Maker (version 2012; Microsoft, Redmond, Washington, USA). Sixteen videos (four per condition) of the original EmpaToM were suitable for teenage participants and were hence included in the EmpaToM-Y. These videos displayed two male and two female Caucasian adults. The corresponding questions were reduced in complexity. Two videos with modified questions from the original EmpaToM served as training trials. Crucially, none of the videos that were used for the EmpaToM-Y appeared in the EmpaToM version applied here.

For each trial of the EmpaToM-Y, we created a multiple-choice question with one correct response option and two distractor options. ToM questions referred to mental state aspects of the narrator, such as thoughts, goals or intentions, that were not explicitly mentioned in the video. Hence, identifying the correct answer to ToM-questions required taking the mental perspective of the previously seen person. Control questions entailed no ToM processes but similarly complex factual reasoning. We devoted considerable effort to ensure a constant level of linguistic demands across the total trials of all four conditions and matched the conditions regarding syntactic complexity and number of words (Table 1). Similarly, the length of the answers was equal across conditions (all *F*s ≤ 1). For the trials of the EmpaToM, the original questions were used.

**Table 1:** Results of analyses on grammatical complexity of the EmpaToM-Y

Dependent variable	Test statistic	*p*-value	Effect size (η^2)^
Number of words	*F*(1, 14) = 0.00	.948	< .01
Frequency of future tense	*F*(1, 14) = 0.08	.787	.01
Frequency of past tense	*F*(1, 14) = 1.91	.189	.12
Number of conditional sentences	*F*(1, 14) = 1.00	.334	.07
Frequency of subordinate clauses	*F*(1, 14) = 0.38	.546	.03

*Note.* For all questions, the number of words, frequencies of future and past tense, number of conditional sentences and the frequency of subordinate clauses were submitted to separate one-way ANOVAs with the within-subject factor condition (neutral-nonToM, neutral-ToM, negative-nonToM, negative-ToM)

#### Procedure

All participants provided written informed consent to the experimental procedures. For the experiment, participants sat 80 cm away from a 60-cm monitor and were provided with a pair of over-ear headphones. The experiment started with a standardized instructions screen, followed by two training trials. For the affect rating, participants were specifically instructed to spontaneously indicate their own emotional state with respect to the video, but to carefully choose their answer to the multiple-choice question. The training block and each of the four test blocks could be started self-paced by pressing the space bar. After the training trials, the participants were given the chance to pose questions to the experimenter, and they had the opportunity to take a break between the blocks. Altogether, it took about 1 hour to complete the experiment.

#### Analyses

Mean absolute affect ratings, mean error rates and mean RTs were submitted to three separate 2 (ToM requirement: ToM, nonToM) × 2 (video valence: negative, neutral) × 2 (task: EmpaToM-Y, EmpaToM) repeated-measures ANOVAs in order to assess (i) whether the EmpaToM-Y was in fact easier, hence eliciting lower error rates and faster responses than the original EmpaToM, and (ii) whether effects of the valence manipulation were comparable across tasks. Post-hoc *t*-tests with Bonferroni correction were performed to resolve ANOVA interaction effects. Additionally, we investigated the effects of video valence and ToM requirements for each of the two instruments individually. The results of these separate ANOVAs can be found in Tables B1–B6 in Appendix B. We report generalized η^2^ as effect size.

For each participant, we calculated individual empathic affect sharing by subtracting the mean affect rating after negative videos from the mean affect rating after neutral videos. Larger values hence indicate a stronger tendency to be influenced by the emotionality of the video and represent greater empathic affect sharing. While difference scores have been criticized for low test-retest reliabilities (Paap & Sawi, 2016), they provide anoption to control for each participant’s baseline and led to reasonable outcomes in previous studies with a similar design (e.g. Bernhardt et al., 2014a). This difference score was calculated separately for trials of the EmpaToM and the EmpaToM-Y, resulting in two values for each participant. We calculated the Pearson correlation between the affect sharing measures of both tasks. Furthermore, we calculated the following Pearson correlations between the EmpaToM and the EmpaToM-Y: (i) mean error rates of ToM questions, (ii) mean error rates of nonToM questions, (iii) mean response times (RTs) for ToM questions and (iv) mean RTs for nonToM questions. RT was defined as the time from question onset until key press.

Following previous studies (Kanske et al., 2015, 2016; Trautwein et al., 2020), we additionally generated composite measures of ToM and nonToM performance by z-transforming the error rates and mean RTs and taking the average of both. Again, we did this separately for the EmpaToM and the EmpaToM-Y to calculate the Pearson correlation between them as well as the partial correlation for the ToM composite values controlling for the nonToM composite values.

Finally, we calculated the internal consistency (Cronbach α) and the item total correlation of each instrument as well as item-specific difficulty and reliability values.

### Results

The datasets generated and analyzed during the current study are available in the Open Science framework repository (DOI: 10.17605/OSF.IO/8Y95B). All stories and questions, as well as an example video of each condition, can be found at the same location. The full video set of the EmpaToM-Y is available on request (DOI: 10.17605/OSF.IO/3RYSN). Both experiments were not pre-registered.

**Fig. 2:**
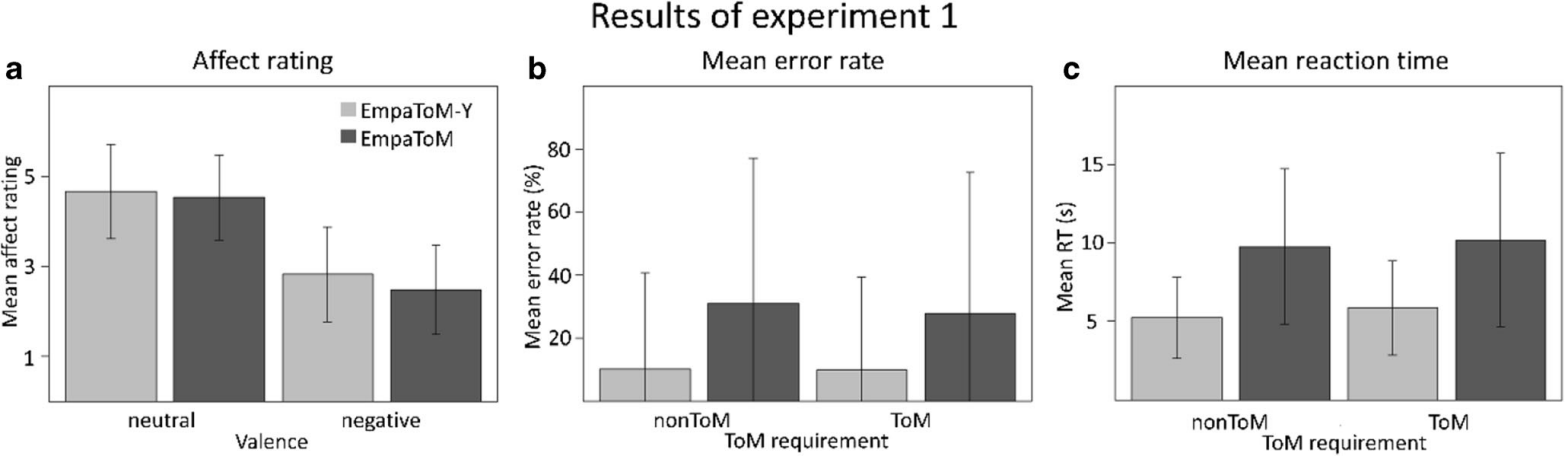
Absolute affect ratings, error rates and RTs per condition in the EmpaToM and the EmpaToM-Y. Note. ToM = Theory of Mind. RT = response time. Error bars represent standard errors. Panel A: Mean affect ratings on a 7-point scale. Panel B: Mean error rates at questions in %. Panel C: Mean response times to questions in seconds

Mean affect ratings, error rates and RTs for each condition of the EmpaToM and the EmpaToM-Y are visualized in Fig. 2 and summarized in Table C1 in Appendix C.

#### Combined ANOVA

##### Affect ratings

In a conjunct analysis of the EmpaToM-Y and the EmpaToM, participants reported significantly more negative affect after videos with negative valence than after neutral videos, reflected in a main effect of video valence (*F*(1, 60) = 351.77, *p* < .001, η^2^ = .85). This pattern is in line with earlier findings and suggests the effectiveness of the empathy induction. Participants also reported more negative affect after nonToM-videos, leading to a main effect of ToM requirement (*F*(1, 60) = 58.60, *p* < .001, η^2^ = .49). The between-subjects factor task (EmpaToM-Y, EmpaToM) was significant (*F*(1, 60) = 101.27, *p* < .001, η^2^ = .63), indicating overall more negative affect in the EmpaToM. This finding likely reflects our decision to remove videos reporting serious negative instances such as abuse and war experiences from the EmpaToM-Y.

There was a significant interaction effect of ToM requirement × video valence (*F*(1, 60) = 18.94, *p* < .001, η^2^ = .24), indicating that the difference between ratings after ToM versus after nonToM videos decreased from neutral to negative, but remained significant (neutral: *t*(121) = 7.25, *p* < .001; negative: *t*(121) = 3.295, *p* < .001). Furthermore, a significant video valence × task interaction effect (*F*(1, 60) = 17.85, *p* < .001, η^2^ = .23) indicates that the difference in affect ratings between the EmpaToM and the EmpaToM-Y was larger after videos with negative valence, but significant in both conditions (neutral: *t*(121) = 3.90, *p* < .001; negative: *t*(121) = 10.46, *p* < .001). No other interactions reached significance (ToM × task: *p* = .223; ToM × video valence × task: *p* = .087).

##### Performance

Participants produced significantly more errors in the original EmpaToM, reflected in a main effect of task (*F*(1, 60) = 276.66, *p* < .001, η^2^ = .82). Hence, as intended, the EmpaToM-Y had reduced levels of difficulty. We also found more errors for neutral videos compared to negative videos, reflected in a main effect of video valence (*F*(1, 60) = 15.80, *p* < .001, η^2^ = .21). The main effect of ToM requirement was not significant (*p* = .134).

We found a significant interaction of ToM requirement × video valence (*F*(1, 60) = 10.23, *p* = .002, η^2^ = .15). This interaction was due to higher error rates for ToM questions, but not for nonToM questions, after neutral compared to after negative videos (ToM: *t*(121) = 4.553, *p* < .001; nonToM: *p* = .448). In addition, there was a significant interaction of video valence × task (*F*(1, 60) = 5.47, *p* = .023, η^2^ = .08), resulting from more errors in in the EmpaToM, but not the EmpaToM-Y, after neutral than after negative videos (EmpaToM: *t*(121) = 3.485, *p* = .004; EmpaToM-Y: *p* = .527). Critically, the ToM requirement × task interaction was not significant (*p* = .214), indicating that the ToM manipulation had similar effects on performance in both tasks.

There was a significant three-way interaction (*F*(1, 60) = 32.21, *p* < .001, η^2^ = .35), resulting from an advantage for neutral videos at nonToM questions in the EmpaToM-Y (*t*(60) = 3.29, *p =* .006), but at ToM questions in the EmpaToM (*t*(60) = 6.567, *p* < .001).

Effects in error rates were paralleled by significantly faster responses for nonToM questions after negative videos and for questions of the EmpaToM-Y, reflected in the significant main effects ToM (*F*(1, 60) = 19.21, *p* < .001, η^2^ = .24), video valence (*F*(1, 60) = 4.20, *p* = .045, η^2^ = .07) and task (*F*(1, 60) = 303.65, *p* < .001, η^2^ = .84), respectively.

The interaction effect of ToM requirement × video valence (*F*(1, 60) = 11.01, *p* < .001, η^2^ = .15) was significant, indicating faster responses to nonToM questions after neutral videos (*t*(123) = −2.92, *p* = .016), but faster responses to ToM questions after negative videos (*t*(121) = 2.56, *p* = .007). A significant video valence × task interaction (*F*(1, 60) = 12.57, *p* = .002, η^2^ = .17) suggested faster responses after negative videos in the EmpaToM (*t*(121) = 2.75, *p* = .020), but no difference in the EmpaToM-Y (*p* = .158). The two-way interaction of ToM requirement × task was not significant (*p* = .839), indicating similar effects of the ToM manipulation across tasks. There was a significant three-way interaction (*F*(1, 60) = 11.41, *p* < .001, η^2^ = .16), indicating that the interaction effect of ToM requirement × video valence was significant only for the EmpaToM with faster responses to ToM questions after negative videos (*t*(60) = 4.34, *p* < . 001).

Taken together, these results indicate effective empathy inductions in both tasks. Also, we successfully reduced task difficulty in the EmpaToM-Y, reflected in both reduced errors and RTs at ToM and nonToM questions. Finally, no main effects of ToM requirements on error rates suggest that overall levels of difficulty were comparable for ToM and nonToM questions in both tasks.

#### Correlations

**Fig. 3:**
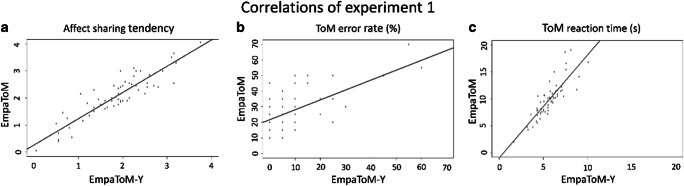
Correlations of affect sharing tendencies as well as errors and RTs in ToM questions between the EmpaToM and the EmpaToM-Y. Note. ToM = Theory of Mind. Panel A: Correlation of affect sharing tendency (difference between ratings after neutral and negative videos) between the EmpaToM and the EmpaToM-Y. Higher values indicate a higher individual tendency for empathic affect sharing. Panel B: Correlation of individual percentages of error rates for ToM questions between the two tasks. Panel C: Correlation of mean response times for questions with ToM requirements between both measures

The correlations of affect sharing tendency and ToM performance are presented in Fig. 3. The mean affect sharing tendency was 2.18 ± .80 (neutral-negative: 4.73–2.55) for the EmpaToM-Y and 2.96 ± 0.87 (neutral-negative: 4.54–2.48) for the EmpaToM. The Pearson correlation between the two sets was *r* = .901 (*p* < .001).

The mean error rate for ToM questions was 10.65 ± 30.85% for the EmpaToM-Y and 28.39 ± 45.11% for the EmpaToM, with a Pearson correlation of *r* = .617 (*p* < .001). The mean error rate for nonToM questions was 11.05 ± 31.36% for the EmpaToM-Y and 31.7 ± 46.54% for the EmpaToM, and the Pearson correlation was *r* = .637 (*p* < .001). When we controlled nonToM on the relationship between ToM responses in the EmpaToM-Y and the EmpaToM, we found a significant partial correlation of *r* = .489 (*p* < .001).

For the EmpaToM-Y, the mean RT for ToM questions was 5.71 ± 1.57 s, whereas it was 10.01 ± 5.56 s for the EmpaToM. The Pearson moment correlation between the tasks was *r* = .849 (*p* < .001). The mean RT for nonToM questions was 5.19 ± 2.37 s for the EmpaToM-Y and 9.61 ± 5.56 s for the EmpaToM, with a Pearson moment correlation of *r* = .628 (*p* < .001). The partial correlation was significant with *r* = 783 (*p* < .001).

The Pearson moment correlation of the composite scores between the EmpaToM-Y and the EmpaToM was *r* = .641 (*p* < .001) for ToM performance and *r* = .494 (*p* < .001) for nonToM questions, with a partial correlation of *r* = .642 (*p* < . 001).

Overall, we found significant, medium to strong correlations of all measures of empathic affect sharing and ToM between the EmpaToM-Y and the EmpaToM, suggesting that our novel task measures the same constructs as the thoroughly validated EmpaToM.

#### Item analyses

The mean error rate of the EmpaToM-Y was 11% both for ToM questions (2–33%) and for nonToM questions (5–39%). The internal consistency of ToM questions was α = .82 (standardized Cronbach α) with an average inter-item correlation of *r* = .19. The correlations between individual items and the total scale ranged from *r* = .21 to *r* = .81. NonToM questions had an internal consistency of α = .88 with an average inter-item correlation of *r* = .27. There was a range of correlations between single items and the total scale of *r* = .16 to *r* = .85.

The mean error rate of the EmpaToM was 29% (5–59%) for ToM questions and 32% (13–59%) for nonToM questions. ToM questions had an internal consistency of α = .57 and an average inter-item correlation of *r* = .06. The correlation of individual items with the total scale ranged between *r* = .07 and *r* = .63. NonToM questions had an internal consistency of α = .67 with an average inter-item correlation of *r* = .09. Single items had a correlation with the total nonToM scale between *r* = .15 and *r =* .58.

In sum, the results indicate strong internal consistency for both the ToM and nonToM scales of our new measure.

### Discussion

Showing strong correlations with an established measure of empathic affect sharing and ToM in adults, experiment 1 demonstrates the validity of our new task (Kanske et al., 2015; Schober et al., 2018). Reduced task demands make the EmpaToM-Y a useful and promising tool for the investigation of social understanding in adolescent samples.

Two findings in particular suggest the validity of assessment of empathic affect sharing in the EmpaToM-Y: First, we found a high correlation between the respective measures of the two instruments. Subjective affect ratings in the EmpaToM are related to performance in other established paradigms for the assessment of empathy (the Socio-affective Video Task; Klimecki et al., 2013) and to neural activation in networks that are commonly associated with empathy (Kanske et al., 2015). This finding is substantiated by the fact that the valence of the videos affected emotion ratings in both instruments. Participants in the present experiment indicated feeling significantly more negative after negative videos compared to after neutral videos. Unsurprisingly, this effect was more pronounced for the EmpaToM, given that this task contains traumatic episodes which are inherently more tragic and hence empathy-inducing than the toned down stories in the EmpaToM-Y. Note that one core goal of our endeavor to create a version of the EmpaToM that would be suitable for adolescents was to exclude traumatic episodes.

We also demonstrate that our new tool is valid for the assessment of ToM by showing adequate correlations with the corresponding measure in the EmpaToM (Schober et al., 2018). This relation was evident both in error rates and RTs for questions that required cognitive perspective taking, and this pattern held even when the correlation between ToM performance in the two measures was controlled for nonToM performance. ToM performance in the EmpaToM has been shown to be related to performance in an established measure of high-level ToM (the Kinderman Imposing Memory task; Kinderman et al., 1998) and the task induced neural activation in regions that are reliably associated with ToM (Kanske et al., 2015). We can thus conclude that the EmpaToM-Y validly measures ToM performance.

Importantly, the results show that the EmpaToM-Y has reduced task demands compared to the EmpaToM. The latter task was designed for adult samples and could be too demanding and tedious for adolescents. Our new task is considerably easier, as evidenced by lower error rates and faster responses, yet it is still capable of revealing inter-individual differences in adults. No item has been answered either correctly or incorrectly by all participants of experiment 1, indicating that every trial of the EmpaToM-Y is suitable to detect inter-individual differences. This pattern is paralleled by convincing internal consistencies of both ToM and nonToM items (Hays & Revicki, 2005) and appropriate item-scale correlations (Piedmont, 2014).

In order to directly target the suitability of our new task for adolescents, experiment 2 applied the EmpaToM-Y to a sample of teenagers aged 14 to 18 years.

## Experiment 2

In experiment 2, we tested the feasibility and appropriateness of the EmpaToM-Y in the intended age group. We employed the task in a group of adolescents and included an established questionnaire for the assessment of socio-cognitive and socio-affective understanding, the German version of the Interpersonal Reactivity Index (i.e. the Saarbrucken Personality Questionnaire, SPQ; Paulus, 2006), for further external validation.

### Method

#### Participants

Forty-three adolescent participants were publicly recruited for experiment 2 and were compensated with payment (10€/hour). Prior to the testing day, all participants were asked to report about mental and neurological disorders as well as about medication. Five participants reported no clinical diagnosis at pre-screening but did so at the test appointment. Due to technical difficulties, the data of a further two participants was missing, leaving 36 participants (14–18 years; mean age = 16.13, SD = 1.40; 24 females) for the final analysis. All of these participants were healthy and unmedicated and had normal or corrected to normal vision. The parents or legal guardians of minor participants provided written informed consent prior to the experiment. Participants of full age provided written informed consent themselves. The present study is compliant with the ethical standards of the 1964 Declaration of Helsinki regarding the treatment of human participants in research and was approved by the local ethics committee.

#### Measures

##### EmpaToM-Y

Only the EmpaToM-Y was employed in experiment 2. This task consisted of three training trials followed by two test blocks of 20 trials each. The trial sequence was similar to experiment 1, but we added a third question at the end of each trial (see Fig. 1). Specifically, participants were asked to rate how familiar they were with the situation that was displayed in the previous video. This served as an indicator of how appropriate the videos are for adolescent samples.

##### Saarbrucken Personality Questionnaire (SPQ)

The SPQ is the revised German version of the Interpersonal Reactivity Index (IRI; Davis, 1980), consisting of the four scales perspective taking (PT), fantasy (FS), empathic concern (EC) and personal distress (PD), with four items per scale. Example items of the IRI can be found in Appendix D. Three of these scales, namely FS, EC and PD, are related to empathy (Paulus, 2006). The remaining scale, PT, is described as the capacity to spontaneously take the psychological perspective of another person and is hence more closely related to ToM. We hypothesized correlations of PT with ToM performance in the EmpaToM-Y, and correlations of the scales FS, EC and PD of the SPQ with empathy tendency in our task. One advantage of the SPQ is the short time it takes to complete the questionnaire: the 16 items are answered in less than 10 minutes, giving us the opportunity to add an external validation measure without excessively prolonging the total duration of the experiment. The SPQ was administered prior to the EmpaToM-Y for half of the participants and after the task for the remaining half (randomized). Altogether, it took about one hour to complete the experiment.

##### Physiological data

In order to test for physiological responses to emotional videos we recorded electrodermal activity and pupillometry during the videos of the EmpaToM-Y. Since we did not find any meaningful effects, the details about data collection and analysis, as well as results, are described in Appendix E.

#### Analyses

As in experiment 1, we calculated separate 2 (video valence: neutral, negative) × 2 (ToM requirement: ToM, nonToM) repeated measures ANOVAs for the following dependent variables: (i) affect ratings, (ii) accuracy, (iii) RTs and (iv) composite scores. We created the latter for ToM and control questions by z-transforming mean correct RTs, defined as the time between question onset and the first key press, and mean amount of error rates, and then taking the average of both. Post-hoc t-tests were performed to resolve ANOVA interaction effects. Furthermore, we tested for correlations between affect ratings, ToM performance and familiarity ratings. We report generalized η^2^ as effect size.

### Results

#### ANOVAs

**Fig. 4:**
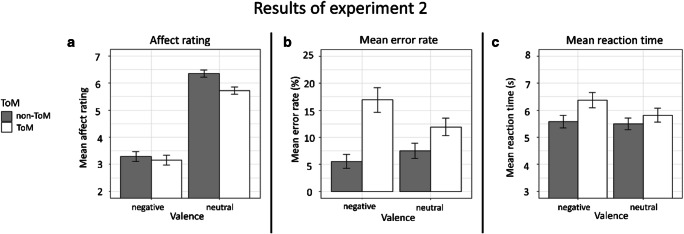
Affect rating and performance results by condition of the EmpaToM-Y in the adolescent sample of experiment 2. *Note.* ToM = Theory of Mind *Panel A*: Mean affect ratings on a 9-point scale. *Panel B*: Mean error rates at questions in %. *Panel C*: Mean response times to questions in seconds

The mean affect ratings as well as mean accuracy rates, RTs and composite scores for each condition are visualized in Fig. 4 and listed in Table C2 in appendix C.

##### Affect ratings

The mean affect sharing tendency was 2.81 (SD = 1.21). Individual affect sharing tendency ranged from −0.25 to 4.80. Adolescents in experiment 2 reported feeling significantly worse after negative videos compared to after neutral videos, reflected in a main effect of video valence (F(1, 35) = 196.09, *p* < .001, η^2^ = .688) and confirming a successful valence manipulation in our task. Ratings were also less positive after videos of the ToM conditions, leading to a main effect of ToM requirement (F(1, 35) = 26.58, *p* < .001, η^2^ = .037). There was a significant interaction of video valence × ToM requirement (F(1, 35) = 12.67, *p =* .001, η^2^ = .016) due to a larger affect sharing tendency after nonToM than after ToM questions (nonToM: *t*(35) = −13.51, *p* < .001; ToM: *t*(35) = −13.02, *p* < .001).

##### Performance

The mean error rates were 14.4% for ToM questions and 6.5% for nonToM questions. Individual error rates for ToM questions ranged between 0.0% and 35.2%, whereas errors ranged between 0% and 14.8% for nonToM questions. For a large majority of 26 participants, ToM questions were more difficult than nonToM questions. ToM and nonToM questions were equally difficult for 6 participants, and for only 4 participants, ToM questions were easier than nonToM questions. Consistently, 10 participants answered all nonToM questions correctly whereas only 2 participants achieved this for ToM questions. One participant performed at ceiling in both conditions.

Hence, in contrast to our findings in adults in experiment 1, adolescents produced significantly more errors for ToM questions than for nonToM questions, reflected in a main effect of ToM requirement (F(1,35) = 33.34, *p* < .001, η^2^ = .135). No main effect of video valence was found (*p* = .381). The video valence × ToM requirement interaction effect was significant due to a larger difference in error rates between ToM and nonToM questions after negative videos (F(1,35) = 4.92, *p* = .033, η^2^ = .029; negative: *t*(35) = 5.04, *p* < .001; neutral: *t*(35) = 2.36, *p* < .001).

Effects in error rates were paralleled by significantly longer RTs to ToM questions than to nonToM questions, reflected in a main effect of ToM requirement (F(1,35) = 17.40, *p* < .001, η^2^ = .036). The valence manipulation induced overall longer response latencies after negative videos (F(1,35) = 13.66, *p* = .001, η^2^ = .012). We found a significant two-way interaction (F(1,35) = 4.82, *p* = .035, η^2^ = .006), reflected in prolonged responses at ToM questions after negative videos (*t*(35) = −4.55, *p* < .001), but not after neutral videos (*p* = .121).

Similar effects emerged in the analysis of the composite scores: Lower scores, indicating better performance, were found for nonToM questions, leading to a main effect of ToM requirement (F(1,35) = 65.27, *p* < .001, η^2^ = .162). A significant main effect of video valence was due to lower scores for questions after neutral videos (F(1,35) = 4.55, *p* = .04, η^2^ = .02). A significant two-way interaction (F(1,35) = 10.49, *p* = .003, η^2^ = .035) indicated once more that the contrast in difficulty was larger after negative videos (negative: *t*(35) = −7.21, *p* < .001; neutral: *t*(35) = −3.48, *p* < .001).

##### Familiarity ratings

The overall familiarity rating was 4.77 with mean item ratings between 1.92 (SD = 1.34) and 7.03 (SD = 1.83) points on a nine-point scale. The SD of items ranged between 1.34 (M = 1.92) and 2.78 (M = 4.06). Mean familiarity ratings and SD of all items are listed in Table F4 in Appendix F.

#### Correlations

More positive valence was reported after videos that were rated as more familiar (*r* = .136, *p* < .001). High familiarity with a situation also was related to faster responses at ToM questions (*r* = -.112, *p* = .005). RTs and error rates for ToM questions were positively related, indicating that questions that were more likely to be answered correctly also were answered faster (*r* = .217, *p* < .001). No other correlations were significant after Bonferroni correction (all *p* > .05). Importantly, ratings of affect were unrelated to performance at ToM questions (accuracy: *p* = .371; RT: *p* = .27).

#### Item analysis

In the adolescent sample, the mean error rate of the EmpaToM-Y was 6.6% (0.0–27.8%) for nonToM questions and 14.4% for ToM questions (0.0–52.8%). Four of the nonToM items and two of the ToM items were always answered correctly but none was unsolvable for all participants. Mean error rates of individual items ranged between 0.0 and 52.8%. The internal consistency of ToM questions was α = .35 (standardized Cronbach α) with an average inter-item correlation of *r* = .03. The correlations between individual items and the total scale ranged from *r* = - .08 to *r* = .66. NonToM questions had an internal consistency of α = .08 with an average inter-item correlation of *r* = .01. There was a range of correlations between single items and the total scale of *r* = .06 to *r* = .52. Taken together, these results indicate that adolescent samples are heterogeneous, producing more variance in ToM and nonToM questions than observed in adults (experiment 1).

#### SPQ

None of the hypothesized correlations between behavioral measures of the EmpaToM-Y and scales of the SPQ were significant (all *p* > .05). In particular, corrected for multiple testing, the scales FS, EC and PD of the SPQ were unrelated to affect sharing tendency of the EmpaToM-Y (FS: *p* = .152; EC: *p* = .162; PD: *p* = .085), and the scale PT was unrelated to ToM accuracy (*p* = .611) and RTs (*p* = .825).

### Discussion

Experiment 2 demonstrates the adequacy of the EmpaToM-Y for the intended age group by showing the general feasibility of the task and sound assessment of empathic affect sharing and ToM with inter-individual variance in adolescents.

The valence manipulation of the EmpaToM-Y induced measurable empathic responses: Participants in experiment 2 indicated feeling significantly more negative after videos with negative valence compared to after neutral videos.

The overall performance in experiment 2 suggests that the EmpaToM-Y is feasible for adolescents. This pattern fits our finding from experiment 1 that the new task is less difficult compared to the original EmpaToM. However, in contrast to results from adults, we found that ToM questions were generally more demanding than control questions for adolescent participants. This effect was evident on all performance measures and substantiates the notion that ToM capacity is still developing during adolescence (Blakemore, 2008; Blakemore et al., 2007; Sebastian et al., 2012; Symeonidou et al., 2020; Wang et al., 2006). While demand differences between ToM and control questions for this age group could constitute a confound in fMRI studies, they offer the opportunity to capture the ToM progression on a behavioral level and contribute to a holistic understanding of social cognition development across the lifespan. Interestingly, once developed, ToM appears to remain relatively stable and even seems to be protected from the overall cognitive decline in the elderly (Reiter et al., 2017).

Given the abovementioned finding that ToM capacity is still developing throughout adolescence, it is reasonable to expect a wide variability in individual ToM performance. As evident in the better performance for nonToM questions, ToM was not yet fully emerged in the present sample of adolescents and, consequently, inter-individual variance was enhanced. Bearing this in mind, it is not surprising that we found relatively low values of internal consistency and item-scale correlations in experiment 2 while experiment 1 showed good internal consistency for ToM performance in adults. Furthermore, prior analyses with adult samples suggest that the items of the EmpaToM are representative for the respective item populations, producing consistent patterns of brain activation for empathy and ToM across item- and participant-wise analyses (Tholen et al., 2020). Given the great conceptual and empirical overlap between the two tasks (see experiment 1), it can be assumed that the same applies for the EmpaToM-Y.

Importantly, and in line with previous findings in adults (Kanske et al., 2016), ToM performance was unrelated to the tendency to share others’ affective states, indicating a successful orthogonal manipulation of empathy and ToM in our new task. This feature allows one to assess the development of both constructs independently from each other. The notion of a conceptual dissociation of empathy and ToM is becoming increasingly popular and has been empirically supported in various domains. First, research suggests independent developmental progress of the two capacities, with ToM preceding empathy in children (Brown et al., 2017), and empathy outliving ToM in older adults (Reiter et al., 2017). Second, a range of mental dysfunctions is known to selectively affect only one aspect of social cognition. The most profound example is a dissociation of social cognitive deficits in ASD and alexithymia (emotion description inability), with an impact of ASD on brain networks related to ToM but not empathy, while the opposite pattern is found for alexithymia (Bernhardt et al., 2014b; Santiesteban et al., 2021). And finally, evidence accumulates that, even in the typically developing brain, cognitive and affective networks in the social brain diverge (Kanske et al., 2015, 2016; Singer, 2006) and can be selectively promoted by specific training modules (Trautwein et al., 2020; Valk et al., 2017). Interestingly, while empathy and ToM are two clearly dissociable tendencies that seem independent in terms of their neural underpinnings and inter-individual variance (for a review, see Stietz et al., 2019), some findings suggest that they interact on an intra-individual level. For instance, empathizing might be prioritized in highly emotional situations, which can hamper ToM performance in this instance (Kanske et al., 2016). For a better understanding of the orchestration of these social capacities within a given person and situation, the simultaneous assessment of these tendencies is critical. Also, for a more thorough understanding of the interplay of empathy and ToM development, a simultaneous assessment of both capacities in different age groups is necessary. The EmpaToM-Y is a promising tool for this endeavor as it allows us to pinpoint inter-individual variance in both components of social understanding in teenagers.

Overall, the familiarity ratings show that the items represent circumstances that adolescents can relate to. There seems to be substantial inter-individual variance in the degree to which participants were familiar with the various situations presented in the videos. This pattern makes the EmpaToM-Y a well-suited task for the assessment of social understanding, because it allows probing these capacities not only in well-known situations, but also when encountering people living in and experiencing circumstances that differ from one’s own. In fact, correlation analyses suggest that high familiarity with a situation might facilitate empathic affect and mental perspective taking. Future studies could use this additional variable to estimate the effect of between-group differences in experiences on social cognition.

We found none of the hypothesized correlations between measures of the EmpaToM-Y and scales of the SPQ. We do not believe, however, that this seriously undermines the validity of our new task. Social cognition is a complex and multifaceted construct and an absence of intercorrelations even between well-established measures is a pattern that has been found before (Dziobek et al., 2006; Osterhaus et al., 2016). Critically, while we assessed actual empathic affect sharing in the EmpaToM-Y, the SPQ is a measure of a person’s conception of her- or himself. Self-reports have been shown to be unrelated to actual behavior in other domains of social cognition, such as altruism (Böckler et al., 2016), and a critical self-reflection of one’s own social cognition capacities could be particularly difficult for adolescents. Furthermore, while the empathy manipulation in our task reflects a psychometric state, the SPQ is a measure of trait empathy (Ze et al., 2014). Finally, a missing relation between the measures could partly be explained by wide-ranging differences in formal aspects of the tasks which have been noted to be critical determinants of the outcome in ToM assessment (Breil & Böckler, 2020) and which should be investigated in future studies with larger samples. As mentioned above, we found strong correlations with an established measure of empathic affect sharing and ToM in experiment 1 (Kanske et al., 2015). Taken together, we believe that, despite the missing link to scales of the SPQ, the EmpaToM-Y is a valid and appropriate tool for the assessment of empathic affect sharing and ToM in adolescent samples.

## General discussion

The present study introduces a novel instrument for the simultaneous assessment of empathic affect sharing and ToM in adolescent samples. In experiment 1, we successfully validated the new task on an established measure of social cognition in a group of adults. In experiment 2, we demonstrated the feasibility of the procedure in the intended age group. The EmpaToM-Y will be a valuable tool in future research and will help to close the gap of knowledge on social cognition between childhood and adulthood.

The valence manipulation of the EmpaToM-Y reliably induced empathic affect sharing in both age groups. Participants indicated feeling significantly more negative after videos with negative valence and experiment 1 revealed a high correlation between affect ratings in our new task and an established measure of empathic affect sharing.

In experiment 1, we found significant correlations of both ToM and nonToM questions between the EmpaToM-Y and an established and thoroughly validated ToM task. While a direct and systematic comparison between experiments 1 and 2 is precarious due to the strong heterogeneity in context variables, there are some noticeable differences that could inspire further research. Our first experiment indicated that both ToM and nonToM questions of our new task were equally demanding for adults. However, adolescents in experiment 2 seemed to find ToM questions more difficult, which is in line with the finding that social cognition is not yet fully emerged in late childhood but instead continues to develop until early adulthood (Blakemore, 2008; Blakemore et al., 2007; Sebastian et al., 2012; Symeonidou et al., 2020; Wang et al., 2006). Note that the very same questions were posed in both experiments of this study, which indicates similar difficulty of ToM and nonToM questions when ToM is fully developed. Our results suggest that this was true already for a small proportion of the adolescent sample, making the EmpaToM-Y a valuable tool to assess the developmental status of ToM beyond childhood. Future studies should apply the present task to larger and representative participant samples to gain a more comprehensive understanding of social affect and social cognition development in adolescents.

While social cognition is under-investigated even in the healthy teenage population, the research demands in adolescents with mental disorders are even higher. In some disorders, including schizophrenia (Bourgou et al., 2016; Li et al., 2017), ASD and Asperger’s syndrome (Kaland et al., 2008), ToM has been investigated more thoroughly even in adolescent samples. For other conditions, such as social anxiety disorder (Öztürk et al., 2020), conduct disorder (Arango Tobón et al., 2017), personality disorders (Sharp et al., 2011) or bipolar disorder (Schenkel et al., 2008), the evidence is still very limited and more research is urgently needed. In all cases, however, systematic investigation of the relationship between social cognition and disease onset and progression are missing. Especially in combination with the EmpaToM (Kanske et al., 2015), the EmpaToM-Y constitutes a promising basis for longitudinal studies assessing empathic affect sharing, ToM and their interplay—an opportunity that, to the best of our knowledge, is given by no other task to the present date. Due to known differences between the sexes in brain development and the incidence of many mental disorders, the role of sex in adolescent social cognition should receive special attention in future research.

In conclusion, we introduce a promising novel task for the assessment of empathic affect sharing and ToM as well as their interaction in adolescents. With its naturalistic setting, the EmpaToM-Y provides the opportunity of capturing inherently interactive capacities in their complexity and studying social understanding in a more realistic and ecologically valid setting. The short implementation duration and stimulating character make the EmpaToM-Y a measure that is particularly suitable for the assessment of social cognition in teenagers. Future studies could use this task to investigate inter-individual variability of socio-cognitive and socio-affective capacities as well as their precursors and outcomes in healthy minors and clinical populations. A first application of the novel task in a healthy sample adds evidence to the notion of an ongoing development of ToM throughout adolescence and a wide range of inter-individual differences in social cognition. This is important groundwork towards a more sophisticated understanding of the developmental trajectory of empathy and ToM beyond childhood and an important extension to our knowledge of social cognition across the lifespan.

## Supplementary Information

ESM 1 (DOCX 39 kb)
