# Supplementary material for: A revised instrument for the assessment of empathy and Theory of Mind in adolescents: Introducing the EmpaToM-Y
Source: Behav Res Methods. 2021 May 4;53(6):2487–501. doi: 10.3758/s13428-021-01589-3 (PMC8613142; doi:10.3758/s13428-021-01589-3)
Supplement: Supplementary file 1 — (DOCX 39 kb) [file 13428_2021_1589_MOESM1_ESM.docx]

**Supplementary material to**

A revised instrument for the assessment of empathy and Theory of Mind in adolescents: Introducing the EmpaToM-Y

Christina Breil^1^, Philipp Kanske ^2,3^, Roxana Pittig^4^, Anne Böckler^1,2^

^1^Leibniz University Hannover, Germany

^2^Max Planck Institute for Human Cognitive and Brain Sciences, Leipzig, Germany

^3^Technische Universität Dresden, Germany

^4^Julius-Maximilians-University of Wuerzburg, Germany

**Corresponding author:**

Christina Breil

Institute of Psychology

Leibniz University of Hannover, Germany

Schloßwender Straße 1

D-30159 Hannover

[breil@psychologie.uni-hannover.de](mailto:breil@psychologie.uni-hannover.de)

Appendix A

**Example stories and corresponding questions of the EmpaToM-Y**

Neutral, nonToM

*“When I come home, my parents are usually still at work. So, I put a pizza into the oven, throw myself on the couch and watch some TV. Except on Thursdays, that’s when I go to basketball training.”*

It is true that Franziska …

a) often eats alone (correct answer)

b) can’t cook (distractor)

c) comes home late (distractor)

Neutral, ToM

*“My big brother is 30 and still living at home. My parents don’t think that’s a problem. They say, he’s gonna move out once he finds a girlfriend. But seriously,* [sarcastic] *him and a girlfriend?!”*

Michael thinks that his brother …

a) probably won’t find a girlfriend (correct answer)

b) would like to move out (distractor)

c) imposes on the kindness of his parents (distractor)

Negative, nonToM

“*I was caught stealing … They literally walked me out handcuffed, in front of all people. What I did was really stupid – but it’s not like I’m a dangerous criminal! Now I’m sentenced to cleaning the youth club.”*

It is true that Alexandra …

a) has to do community service (correct answer)

b) doesn’t have a lot of money (distractor)

c) is stealing in the store on a regular basis (distractor)

Negative, ToM

“*One of my classmates comes from a very poor family. She never has anything to eat with her. Recently, I offered her something from my lunch bag, and she told me she was on a diet. But she is super skinny! I felt so sorry for her…”*

Michael thinks that his classmate

a) tries to keep her poverty a secret (correct answer)

b) is anorectic (distractor)

c) is lying (distractor)

Appendix B

**Seperate ANOVAs of the EmpaToM-Y and the EmpaToM**

**Table B1**

*Affect sharing ratings of the EmpaToM-Y*

| Factor | Test statistic | *p-*value | η^2^_generalized_ |
| --- | --- | --- | --- |
| Main effect of ToM requirement | *F*(1,60) = 26.01 | < .001 | .30 |
| Main effect of video valence | *F*(1,60) = 323.76 | < .001 | .84 |
| Interaction of ToM requirement × video valence | *F*(1,60) = 17.42 | < .001 | .22 |

Post-hoc one-sided t-test (Bonferroni-corrected)

Neutral: *t*(60) = 5.07, *p* < .001

Negative: *p* > .05

**Table B2**

*Error rates of the EmpaToM-Y*

| Factor | Test statistic | *p-*value | η^2^_generalized_ |
| --- | --- | --- | --- |
| Main effect of ToM requirement | *F*(1,60) =.18 | .669 | <.01 |
| Main effect of video valence | *F*(1,60) = 2.81 | .099 | .04 |
| Interaction of ToM requirement × video valence | *F*(1,60) = 6.55 | .013 | .10 |

Post-hoc one-sided t-test (Bonferroni-corrected)

ToM: *p* > .05

nonToM: *t*(60) = 3.29, *p* = .0016

**Table B3**

*RTs of the EmpaToM-Y*

| Factor | Test statistic | *p-*value | η^2^_generalized_ |
| --- | --- | --- | --- |
| Main effect of ToM requirement | *F*(1,60) = 29.36 | < .001 | .33 |
| Main effect of video valence | *F*(1,60) = 3.35 | .072 | .05 |
| Interaction of ToM requirement × video valence | *F*(1,60) = 0.19 | .665 | .01 |

**Table B4**

*Affect sharing ratings of the EmpaToM*

| Factor | Test statistic | *p-*value | η^2^_generalized_ |
| --- | --- | --- | --- |
| Main effect of ToM requirement | *F*(1,60) = 44.53 | < .001 | .43 |
| Main effect of video valence | *F*(1,60) = 344.50 | < .001 | .85 |
| Interaction of ToM requirement × video valence | *F*(1,60) = 5.61 | .021 | .09 |

Post-hoc one-sided t-test (Bonferroni-corrected)

Neutral: *t*(60) = 5.15, *p* < .001

Negative: *t*(60) = 4.51, *p* < .001

**Table B5**

*Error rates of the EmpaToM*

| Factor | Test statistic | *p-*value | η^2^_generalized_ |
| --- | --- | --- | --- |
| Main effect of ToM requirement | *F*(1,60) = 2.63 | .110 | .04 |
| Main effect of video valence | *F*(1,60) = 14.85 | < .001 | .20 |
| Interaction of ToM requirement × video valence | *F*(1,60) = 27.45 | < .001 | .31 |

Post-hoc one-sided t-test (Bonferroni-corrected)

ToM: *t*(60) = 6.57, *p* <.001

nonToM: *p* > .05

**Table B6**

*RTs of the EmpaToM*

| Factor | Test statistic | *p-*value | η^2^_generalized_ |
| --- | --- | --- | --- |
| Main effect of ToM requirement | *F*(1,60) = 6.08 | .017 | .09 |
| Main effect of video valence | *F*(1,60) = 9.54 | .003 | .14 |
| Interaction of ToM requirement × video valence | *F*(1,60) = 12.26 | .001 | .17 |

Post-hoc one-sided t-test (Bonferroni-corrected)

ToM: *t*(60) = 4.34, *p* < .001

nonToM: *p* > .05

Appendix C

**Results of experiments 1 and 2**

**Table C1**

Mean affect sharing ratings, error rates and RTs for all conditions of the EmpaToM and the EmpaToM-Y in experiment 1

| Task | Video valence | ToM-requirement | Mean affect sharing (SD) | Mean error rate in % (SD) | Mean RT in s (SD) |
| --- | --- | --- | --- | --- | --- |
| EmpaToM-Y | neutral | nonToM | 4.85 (±0.98) | 13.39 (±34.07) | 5.16 (±2.29) |
| EmpaToM-Y | neutral | ToM | 4.5 (±1.08) | 10.33 (±30.44) | 5.67 (±2.77) |
| EmpaToM-Y | negative | nonToM | 2.84 (±1.08) | 8.71 (±28.22) | 5.33 (±2.84) |
| EmpaToM-Y | negative | ToM | 2.81 (±1.02) | 10.97 (±31.27) | 6.09 (±3.16) |
| EmpaToM | neutral | nonToM | 4.68 (±0.08) | 30 (±45.86) | 9.50 (±4.75) |
| EmpaToM | neutral | ToM | 4.38 (±0.98) | 36.78 (±48.25) | 10.87 (±6.17) |
| EmpaToM | negative | nonToM | 2.56 (±0.95) | 33.39 (±47.19) | 10.11 (±5.21) |
| EmpaToM | negative | ToM | 2.41 (±1.03) | 20 (±40.03) | 9.50 (±4.74) |

*Note*. ToM = Theory of Mind; RT = reaction time.

**Table C2**

*Mean affect sharing ratings and familiarity, mean accuracy rates and mean RTs for each condition of experiment 2*

| Video valence | ToM requirement | M (SD) affect sharing rating | M (SD) accuracy (%) | M (SD) reaction time (ms) | M (SD) composite |
| --- | --- | --- | --- | --- | --- |
| negative | nonToM | 3.29 (±1.11) | 94.44 (±7.72) | 5689.4 (±1364.5) | -0.30 (±0.64) |
| negative | ToM | 3.16 (±1.09) | 83.06 (±13.69) | 6665.2 (±1710.2) | 0.48 (±0.69) |
| neutral | nonToM | 6.35 (±0.79) | 92.50 (±8.41) | 5676.2 (±1287.8) | -0.25 (±0.56) |
| neutral | ToM | 5.73 (±0.80) | 88.06 (±9.80) | 6171.7 (±1642.8) | 0.07 (±0.63) |

*Note.* ToM = Theory of Mind. RT = reaction time. The composite measure is a combination of RT and error rate.

Appendix D

**Saarbrucken Personality Questionnaire (SPQ)**

Answer format

Never seldom sometimes often always

1 (--) 2 (-) 3 (o) 4 (+) 5 (++)

The SPQ is the German version of the Interpersonal Reactivity Index (IRI) by Davis (1980).

Example items of the original IRI by Davis (1980)

*Empathic concern*

I often have tender, concerned feelings for people less fortunate than me.

*Fantasy*

After seeing a play or movie, I have felt as though I were one of the characters.

*Personal distress*

In emergency situations, I feel apprehensive and ill-at-ease.

*Perspective taking*

When I'm upset at someone, I usually try to "put myself in his shoes" for a while.

**References**

Davis, H. (1980). Measuring individual differences in empathy: Evidence for a multidimensional approach. *Journal of Personality and Social Psychology*, *44*(1), 113–132.

Appendix E

**Physiological data**

***Rationale***

To test whether empathic responses were reflected in physiological arousal, we recorded electrodermal activity (EDA) and pupillometry during the presentation of the videos. We expected higher skin conductance responses (SCRs) and pupil dilation during negative compared to neutral videos.

***Procedure***

Participants were seated 80cm away from a 69-cm monitor and placed their head in a chin rest. A pair of disposable snap electrodes was placed on the palm of their left hand for EDA measurement while their right hand rested on a button box. This set-up allowed the participants to complete the EmpaToM-Y while holding their head and their left hand completely still in order to minimize movement artefacts in the physiological data.

The task and calibration procedure were carefully explained to each participant by the experimenter. The task started with a standardized instruction screen, followed by a nine-point calibration and three training trials. After the training block, the participants had once again the chance to pose questions to the experimenter before the first test block started. The experimental flow was similar to experiment 1 but consisted of 40 trials only, randomly divided into two test blocks with an optional break in between. The SPQ was administered prior to the EmpaToM-Y for half of the participants and after the task for the remaining half (randomized). Altogether, it took about one hour to complete the experiment.

**Pupillometry.** The pupillometry during the videos was tracked with an EyeLink 1000 Plus eye tracker (SR Research, Ontario, Canada) and analyzed with the corresponding software (Data Viewer version 3.2). Calibration was performed prior to training and each test block. Additionally, a drift check was performed before every trial and calibration was initiated when the accuracy of the calibration parameters was too low.

**EDA measurement.** Exosomatic EDA was measured with direct current, using a constant voltage system (MP160 acquisition system, BIOPAC Systems Inc., Goleta, USA). A pair of disposable Ag/AgCl electrodes (size: 27mm × 36mm × 1.5mm) was placed on the thenar and hypothenar sites of the palms of the left hand of each participant and connected to an external voltage of 0.5V. An isotonic 0.05 molar NaCl electrode paste was used as contact electrolyte.

The data of each participant was analyzed individually using ACQKnowledge (BIOPAC Systems Inc., Goleta, USA) according to the following protocol: First, the data signal was smoothed and amplified. Second, an event-related EDA analysis was performed, deriving the phasic waveform from tonic and locating SCRs within a time window of 20s after video onset in each trial. This timeframe allows for a detection of all SCRs that could have been caused by the stimulus, even when triggered at the end of the video (i.e. 10 – 15s after onset). We chose a threshold of 0.1µS and SCRs under 10% of the maximum curve were rejected (Braithwaite et al., 2013).

***Analyses***

Physiological correlates of arousal were measured by means of ER-SCR amplitude and pupillometry. For both dependent variables, we analyzed the main effect of video valence by means of repeated measures ANOVAs to test for differences in physiological arousal between the video conditions. Furthermore, we calculated the correlation between affect sharing ratings and (i) ER-SCR amplitude and (ii) maximum pupil size both on a trial level (absolute ratings) and on an individual level (tendency: negative-neutral) to test for relations between subjective and physiological empathic affect sharing. All t-tests and correlations were corrected for multiple testing using the Bonferroni formula.

***Results and discussion***

There was no difference in ER-SCR amplitude (*p* = .146) or pupil size (*p* = .362) between neutral and negative videos. However, individual affect sharing tendency, calculated as the mean affect sharing rating after positive videos minus after emotional videos, was negatively related to the ER-SCR amplitude difference between neutral and emotional videos (*r* = -.062, *p* = .04). Albeit consistent with our hypothesis, this effect was too small to indicate that participants who were more empathic on a subjective level also felt more arousal on a physiological level. Furthermore, affect sharing tendency was unrelated to maximum pupil size difference (*p* = .982). On a trial level, absolute affect sharing ratings were not related to maximum pupil size (*r* = -.053, *p* = .08) and affect sharing ratings showed no correlation to ER-SCR amplitude on a trial level (*p* = .603).

While some studies reported a connection between empathy for pain and physiological indicators of arousal, including pupil dilation and SCR (e.g. Hein et al., 2011; Krebs, 1975; Michalska et al., 2013; van Zonneveld et al., 2017), other studies found no relationship between SCR and affect sharing measures such as in our task (Kanske et al., 2015). Even though it cannot be ruled out that the missing link between both measures in our study is due to small effect sizes and/or power issues, it seems likely that only some forms of empathy, such as observing pain, are mirrored by changes in pupil dilation and SCR.

**References**

Braithwaite, J. J., Watson, D. G., Jones, R., & Rowe, M. (2013). A guide for analysing electrodermal activity (EDA) & skin conductance responses (SCRs) for psychological experiments. *Psychophysiology*, *49*(1), 1017–1034.

Hein, G., Lamm, C., Brodbeck, C., & Singer, T. (2011). Skin Conductance Response to the Pain of Others Predicts Later Costly Helping. *PLoS ONE*, *6*(8), e22759. https://doi.org/10.1371/journal.pone.0022759

Kanske, P., Böckler, A., Trautwein, F.-M., & Singer, T. (2015). Dissecting the social brain: Introducing the EmpaToM to reveal distinct neural networks and brain–behavior relations for empathy and Theory of Mind. *NeuroImage*, *122*, 6–19. https://doi.org/10.1016/j.neuroimage.2015.07.082

Krebs, D. (1975). Empathy and altruism. *Journal of Personality and Social Psychology*, *32*(6), 1134.

Michalska, K. J., Kinzler, K. D., & Decety, J. (2013). Age-related sex differences in explicit measures of empathy do not predict brain responses across childhood and adolescence. *Developmental Cognitive Neuroscience*, *3*, 22–32. https://doi.org/10.1016/j.dcn.2012.08.001

van Zonneveld, L., Platje, E., de Sonneville, L., van Goozen, S., & Swaab, H. (2017). Affective empathy, cognitive empathy and social attention in children at high risk of criminal behaviour. *Journal of Child Psychology and Psychiatry*, *58*(8), 913–921. https://doi.org/10.1111/jcpp.12724

Appendix F

**Table F4**

*Mean, SD and range of familiarity ratings of all items of the EmpaToM-Y in an adolescent sample group (N = 36)*

| Item number | Valence | ToM requirement | mean | sd | min | max |
| --- | --- | --- | --- | --- | --- | --- |
| 1.1 | neutral | nonToM | 6.50 | 1.28 | 4 | 9 |
| 1.2 | neutral | ToM | 5.11 | 1.35 | 3 | 9 |
| 1.3 | negative | nonToM | 5.08 | 1.76 | 2 | 9 |
| 1.4 | negative | ToM | 4.50 | 1.77 | 3 | 9 |
| 2.1 | neutral | nonToM | 7.19 | 1.39 | 3 | 9 |
| 2.2 | neutral | ToM | 5.03 | 1.36 | 3 | 9 |
| 2.3 | negative | nonToM | 3.83 | 1.44 | 1 | 8 |
| 2.4 | negative | ToM | 2.50 | 1.42 | 1 | 7 |
| 3.1 | neutral | nonToM | 5.50 | 1.44 | 3 | 9 |
| 3.2 | neutral | ToM | 5.17 | 1.42 | 2 | 9 |
| 3.3 | negative | nonToM | 3.00 | 1.39 | 1 | 7 |
| 3.4 | negative | ToM | 3.11 | 1.47 | 1 | 8 |
| 4.1 | neutral | nonToM | 6.44 | 1.46 | 4 | 9 |
| 4.2 | neutral | ToM | 5.97 | 1.23 | 4 | 9 |
| 4.3 | negative | nonToM | 3.56 | 1.18 | 2 | 8 |
| 4.4 | negative | ToM | 3.75 | 1.46 | 2 | 8 |
| 5.1 | neutral | nonToM | 6.86 | 1.25 | 5 | 9 |
| 5.2 | neutral | ToM | 6.67 | 1.39 | 3 | 9 |
| 5.3 | negative | nonToM | 3.36 | 1.31 | 1 | 8 |
| 5.4 | negative | ToM | 3.92 | 1.42 | 2 | 8 |
| 6.1 | neutral | nonToM | 6.64 | 1.36 | 4 | 9 |
| 6.2 | neutral | ToM | 6.06 | 1.19 | 4 | 8 |
| 6.3 | negative | nonToM | 2.33 | 1.47 | 1 | 8 |
| 6.4 | negative | ToM | 2.22 | 1.22 | 1 | 7 |
| 7.1 | neutral | nonToM | 5.89 | 1.45 | 3 | 9 |
| 7.2 | neutral | ToM | 6.19 | 1.33 | 3 | 8 |
| 7.3 | negative | nonToM | 2.25 | 1.44 | 1 | 8 |
| 7.4 | negative | ToM | 2.50 | 1.32 | 1 | 8 |
| 8.1 | neutral | nonToM | 6.50 | 1.34 | 3 | 9 |
| 8.2 | neutral | ToM | 5.36 | 1.61 | 2 | 9 |
| 8.3 | negative | nonToM | 3.64 | 1.44 | 1 | 8 |
| 8.4 | negative | ToM | 3.25 | 1.54 | 1 | 9 |
| 9.1 | neutral | nonToM | 6.83 | 1.52 | 2 | 9 |
| 9.2 | neutral | ToM | 4.72 | 1.21 | 2 | 8 |
| 9.3 | negative | nonToM | 3.31 | 1.69 | 1 | 8 |
| 9.4 | negative | ToM | 2.94 | 1.24 | 1 | 7 |
| 10.1 | neutral | nonToM | 5.11 | 1.43 | 2 | 8 |
| 10.2 | neutral | ToM | 7.03 | 1.75 | 2 | 9 |
| 10.3 | negative | nonToM | 2.53 | 1.58 | 1 | 8 |
| 10.4 | negative | ToM | 2.92 | 1.34 | 1 | 8 |
